# Coexistence of ANCA-associated glomerulonephritis and membranous nephropathy: a scoping review

**DOI:** 10.1007/s10157-026-02904-y

**Published:** 2026-06-10

**Authors:** Ryosuke Saiki, Kan Katayama, Mutsuki Mori, Kaoru Dohi

**Affiliations:** Department of Cardiology and Nephrology, Mie University Graduate School of Medicine, 2-174 Edobashi, Tsu, Mie 514-8507 Japan

**Keywords:** ANCA-associated glomerulonephritis, Membranous nephropathy, Myeloperoxidase, Proteinase-3, A scoping review

## Abstract

**Background:**

Coexistence of anti-neutrophil cytoplasmic antibody -associated glomerulonephritis (ANCA-GN) and membranous nephropathy (MN) is rare. This scoping review mapped evidence on clinicopathologic features, phenotypic heterogeneity, and reporting gaps relevant to future rigorous phenotype-specific comparative studies.

**Methods:**

Comprehensive searches of PubMed, Web of Science, and CENTRAL from inception to April 16, 2026 identified original studies of patients with coexisting ANCA-GN and MN. Two reviewers independently extracted study and participant characteristics, renal and systemic findings, serologic and antigen data, associated conditions, treatments, outcomes, phenotype categories, and temporal relationships. Individual-level cases were assigned using a prespecified phenotype framework.

**Results:**

Seventy studies (140 patients) were included, comprising 113 individually charted cases and one aggregate-only cohort of 27 patients. Among individual-level cases, median age was 60 years; median serum creatinine, 2.75 mg/dL; and median proteinuria, 3.7 g/day or g/g creatinine (g/gCr). The overlap was phenotypically heterogeneous, including 66 likely myeloperoxidase (MPO)-associated/ANCA-GN-related cases, 10 likely primary-MN-like overlap cases, 31 secondary/associated cases, and 6 unclassifiable cases. Temporal relationship was ascertainable for all 113 cases: 101 (89.4%) had concurrent onset (≤ 6 months), 7 (6.2%) had MN preceding ANCA-GN, and 5 (4.4%) had ANCA positivity preceding MN. The evidence base was constrained by case-report predominance, selective antigen testing, sparse longitudinal biomarker/follow-up data, and nonuniform outcome reporting.

**Conclusions:**

Coexisting ANCA-GN and MN is a rare, clinically important, and heterogeneous spectrum rather than a single pooled entity. Future studies should use phenotype-specific designs with standardized antigen testing, crescent reporting, longitudinal ANCA assessment, consistent outcome definitions, and explicit follow-up.

**Supplementary Information:**

The online version contains supplementary material available at https://doi.org/10.1007/s10157-026-02904-y.

## Introduction

Anti-neutrophil cytoplasmic antibody (ANCA)-associated vasculitis is an autoimmune condition marked by inflammation of small blood vessels [1]. In vitro research shows that ANCA binding to these targets activates neutrophils, causing cytokine production, degranulation, neutrophil extracellular trap formation, and reactive oxygen species production [2]. ANCA-associated vasculitis can affect a broad spectrum of organs [1]. Kidney involvement is one of its major organ manifestations and is clinically important because active ANCA-associated glomerulonephritis (ANCA-GN) can lead to rapidly progressive kidney dysfunction and irreversible kidney failure if not recognized and treated promptly [3].

In ANCA-GN, extracapillary crescents constitute the predominant renal lesion, occurring in approximately 90% of cases [4]. The typical biopsy reveals necrotizing and crescentic glomerulonephritis with little or no immune-complex deposition, which is interpreted as pauci-immune glomerular injury. Nevertheless, immune-complex deposits have been described in a subset of cases, and membranous nephropathy (MN) -like lesions have occasionally been reported in association with ANCA-GN. MN is a subepithelial immune-complex glomerular disease and is classified according to target antigens, including phospholipase A2 receptor (PLA2R), thrombospondin type-1 domain-containing 7 A (THSD7A), and other recently recognized antigens [5, 6]. Notably, immunohistochemical studies have demonstrated myeloperoxidase (MPO) deposition within the subepithelial deposits along the glomerular basement membrane in a subset of cases with coexisting ANCA-GN and MN, raising the possibility that MPO released from activated neutrophils acts as a planted antigen driving the membranous lesion [7–9].

Accumulating reports suggest that coexisting ANCA-GN and MN does not represent a single disease entity but rather a heterogeneous clinicopathologic spectrum that includes likely MPO-associated or ANCA-GN-related MN, primary-MN-like coincidental cases, secondary or associated forms, and unclassifiable cases. Distinguishing these possibilities is clinically relevant because the same biopsy-level coexistence may reflect different diagnostic problems and may require different interpretation of antigen testing, associated conditions, treatment timing, and prognosis. This heterogeneity is compounded by inconsistent reporting across studies, including differences in antigen testing, case definitions, outcome measures, timing of assessment, and follow-up duration. These limitations make it difficult to compare treatment responses or prognosis across published cases. In view of these issues, this scoping review will map the available evidence, summarize the heterogeneity of coexisting ANCA-GN and MN, and identify data gaps relevant to future comparative studies. This approach was chosen because the literature is sparse, heterogeneous, and dominated by case reports and small case series, making evidence mapping more appropriate than pooled quantitative synthesis.

## Materials and methods

### Study design and protocol

 .

This scoping review followed Joanna Briggs Institute (JBI) methods, is reported according to PRISMA-ScR guidelines [10, 11], and was preregistered with the Open Science Framework (https://osf.io/k4jch/). We examined reported clinical presentations, clinicopathologic phenotypes, treatment patterns, and gaps relevant to future comparative studies. The review questions were: (1) what are the clinical presentations, clinicopathologic phenotypes, and treatment patterns of coexisting ANCA-GN and MN? and (2) what are the main gaps in the available literature, and what information would be required for future comparative studies of coexisting ANCA-GN and MN? 

### Eligibility criteria

Using the Population-Concept-Context framework, we included original studies of patients with coexisting ANCA-GN and MN without restrictions on age, sex, geography, language, or setting. We reviewed the existing literature on the clinicopathologic features, treatment strategies, and outcomes of coexisting ANCA-GN and MN. The review specifically focused on phenotype heterogeneity, including likely MPO-associated or ANCA-GN-related MN, likely coincidental primary MN with ANCA-GN, secondary or associated MN, and unclassifiable cases. We also evaluated the feasibility of future comparative studies by clarifying gaps in phenotype definition, antigen testing, severity assessment, follow-up, and outcome reporting. Eligible reports had to document ANCA seropositivity by immunofluorescence or antigen-specific assay; incomplete antigen detail was accepted if MPO-ANCA, proteinase 3-ANCA, perinuclear ANCA, or cytoplasmic ANCA positivity was explicit. We excluded reports describing the overlap without documented ANCA seropositivity, as well as reviews and conference abstracts. 

### Information sources and search strategy

We searched PubMed, Web of Science, and CENTRAL from inception to April 16, 2026, and screened the reference lists of included studies. The search combined terms for membranous nephropathy or membranous glomerulopathy with terms for ANCA-associated vasculitis, ANCA-GN, microscopic polyangiitis, granulomatosis with polyangiitis, eosinophilic granulomatosis with polyangiitis, and ANCA serology. We included published randomized controlled trials, crossover trials, cluster-randomized trials, quasi-randomized trials, non-randomized trials, observational studies, case reports, and case series. No language, age, sex, geographic, or setting restrictions were applied. Database-specific search strategies are provided in Online Resource 1. 

### Study selection

After deduplication, two reviewers independently screened titles/abstracts and full texts, resolving disagreements by discussion or a third reviewer. Reasons for exclusion were recorded at the full-text stage to support transparent reporting of the selection process.

### Data extraction

We used a standardized form and two independent reviewers to chart study- and individual-level data on renal severity/pathology, systemic manifestations, serologic and antigen findings, associated conditions, treatments, outcomes, phenotype, and temporal relationship. We also recorded whether data were available at the individual-patient level or only at the aggregate-study level. Extracted variables included age, sex, country, comorbidities, serum creatinine, proteinuria, hematuria, rapidly progressive glomerulonephritis presentation, crescent presence and percentage, extrarenal manifestations, ANCA type and titer, PLA2R and THSD7A status, IgG subclass findings, MPO deposition in membranous lesions, associated malignancy or autoimmune disease, drug or other triggers, withdrawal of suspected drugs or triggers, immunosuppressive treatment, plasma exchange, remission as defined by the original authors, kidney outcome, and follow-up. Missing or unclear variables were coded as not reported rather than inferred unless explicitly stated in the original report. Where data permitted, individual-level cases were assigned to likely ANCA-GN-related MN, likely primary-MN-like overlap with ANCA-GN, secondary / associated MN, or unclassifiable using a prespecified phenotype framework (Online Resource 2). Phenotype assignment and temporal classification were performed at the individual-case level and discrepancies were resolved by reviewer discussion using the prespecified framework. Temporal relationship was used as supportive information and was not used as the sole determinant of phenotype assignment. The aggregate-only cohort was summarized separately and was not merged into phenotype-specific individual-level analyses because patient-level phenotype assignment was not possible. Temporal relationship was classified according to the interval between renal biopsy and the first documented ANCA positivity as concurrent onset ( < = 6 months), MN preceding ANCA-GN (> 6 months), ANCA preceding MN (> 6 months), or unascertainable.

### Data synthesis

Findings were synthesized narratively with structured tables and figures, including the PRISMA-ScR flow diagram and Online Resources 2–4. We summarized the evidence base separately at the study level and, where available, at the individual-patient level. Because the evidence base consisted mainly of case reports and small case series with heterogeneous phenotype definitions, selective antigen testing, inconsistent outcome definitions, and variable follow-up, we did not perform meta-analysis or formal comparative treatment-effect estimation. In keeping with scoping review methodology, no formal risk-of-bias or certainty-of-evidence assessment was performed; instead, we focused on mapping the available evidence and identifying minimum reporting elements needed for future comparative studies. Reporting gaps were summarized with attention to phenotype definition, antigen testing, severity assessment, outcome definitions, timing of outcome assessment, follow-up duration, and longitudinal biomarker reporting. We also considered the feasibility of future comparative studies based on the completeness and consistency of available case-level data.

## Results

### Study selection

**Fig. 1:**
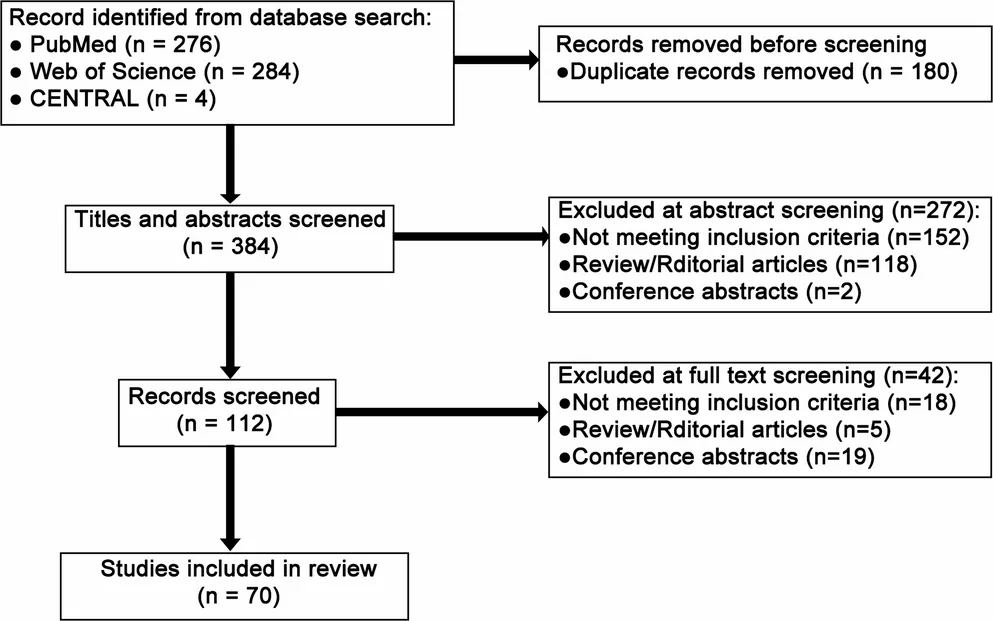
PRISMA-ScR flow diagram of the study selection process The diagram illustrates the flow of information through the different phases of the scoping review, from the initial identification of records in the databases to the final inclusion of studies. The updated search and screening process culminated in 70 included studies in the narrative synthesis

The updated search and selection process is shown in Fig. 1. After deduplication and two-stage screening, 70 studies were included. Because the evidence base was dominated by case reports and small case series, the synthesis is descriptive rather than pooled.

### Characteristics of the evidence base

The literature comprised 140 patients with coexisting ANCA-GN and MN: 113 individually charted cases and one aggregate-only cohort of 27 patients. The individual dataset and references to included individual-level reports are provided in Online Resource 5, and the aggregate-only cohort by Zou et al. is provided in Online Resource 6 [12]. Among individual-level cases, 49/113 (43.4%) were reported from Asia, 41/113 (36.3%) from North America, 20/113 (17.7%) from Europe, and 3/113 (2.7%) from other regions; Japan accounted for 29 cases and the United States for 37. 

### Phenotypic heterogeneity and temporal relationship

Using the prespecified phenotype framework (Online Resource 2), 66/113 (58.4%) individual-level cases were classified as likely ANCA-GN-related MN, 10/113 (8.8%) as likely primary-MN-like overlap with ANCA-GN, 31/113 (27.4%) as secondary / associated MN, and 6/113 (5.3%) as unclassifiable. Temporal relationship was ascertainable for all 113 cases: 101/113 (89.4%) had concurrent onset, 7/113 (6.2%) had MN preceding ANCA-GN, and 5/113 (4.4%) had ANCA positivity preceding MN. 

### Clinicopathologic features and targeted descriptive comparisons

Clinicopathologic features are summarized in Online Resource 3. Median age at diagnosis was 60 (10–88) years, 46/113 (40.7%) were female, median serum creatinine was 2.75 (0.13–14.5) mg/dL, and median proteinuria was 3.7 (0.2–37.8) g/day or g/g creatinine (g/gCr). Hematuria was reported in 81/83 (97.6%), crescents in 97/109 (89.0%), and the median percentage of glomeruli with crescents was 33 (4–92)%.

In targeted descriptive comparisons, MPO-ANCA was present in 75/112 (67.0%), PR3-ANCA in 19/112 (17.0%), and double-positive ANCA in 9/112 (8.0%). Phospholipase A2 receptor (PLA2R) status was assessed in 31/113 (27.4%) cases and was positive in 10/31 (32.3%); thrombospondin type-1 domain-containing 7 A (THSD7A) was assessed in 1/113 (0.9%) case and was negative. Immunoglobulin G (IgG) subclass data were reported in 19/113 (16.8%) cases, and malignancy in 4/99 (4.0%). Among PLA2R-assessed cases, median proteinuria was 6.74 g/day or g/gCr in PLA2R-positive cases versus 2.57 in PLA2R-negative cases, whereas crescents were frequent in both groups (8/9 and 19/21, respectively); a descriptive subgroup comparison is provided in Online Resource 7. Rapidly progressive glomerulonephritis (RPGN) was reported in 75/88 (85.2%) cases, but RPGN-specific outcome comparison was not feasible because follow-up and remission definitions were inconsistent. 

### Treatment patterns and kidney outcomes across categories

Among cases with reported treatment data, glucocorticoids were used in 95/100 (95.0%), cyclophosphamide in 58/100 (58.0%), rituximab in 10/100 (10.0%), plasma exchange in 13/100 (13.0%), and other immunosuppressive agents in 27/100 (27.0%). Among cases with evaluable kidney outcomes, end-stage kidney disease (ESKD) occurred in 24/97 (24.7%) overall: 17/60 (28.3%) likely ANCA-GN-related cases, 1/6 (16.7%) likely primary-MN-like overlap cases, 5/27 (18.5%) secondary / associated cases, and 1/4 (25.0%) unclassifiable cases. The aggregate-only cohort reported severe kidney involvement and ESKD in 48.1% (Online Resource 6). 

### Data gaps relevant to future comparative studies

The minimum reporting elements for future comparative studies are summarized in Online Resource 4. At the study level, baseline ANCA type/titer was reported in 55/70 (78.6%) studies, percent crescents in 50/70 (71.4%), and PLA2R/THSD7A status in 23/70 (32.9%). Longitudinal ANCA follow-up was reported in 18/70 (25.7%) studies, whereas explicit phenotype frameworks were reported in 0/70 and remission definitions in 3/70 (4.3%); assessment time points and follow-up duration were insufficiently reported. Comparative inference remained limited by nonuniform phenotype definition, selective antigen testing, and sparse longitudinal biomarker and outcome reporting. In particular, limited reporting of MPO deposition in membranous lesions, selective PLA2R/THSD7A testing, and inconsistent outcome time points prevented robust comparison of putative MPO-associated, primary-MN-like, and secondary / associated phenotypes.

## Discussion

This scoping review mapped 70 studies involving 140 patients with coexisting ANCA-GN and MN and showed that this overlap is uncommon, clinically severe, and heterogeneous rather than a single pooled entity.

An important finding of this review is that coexisting ANCA-GN and MN should not be treated as a single clinicopathologic entity. The literature includes likely MPO-associated / ANCA-GN-related MN, likely primary-MN-like overlap, and secondary / associated forms, indicating that multiple clinical pathways are represented within the published cases. A few reports showed MPO-positive staining in membranous deposits [10, 11], compatible with an MPO-associated mechanism in some cases, but antigen-defined primary-MN-like overlap, drug-related cases, lupus-related cases, rheumatoid arthritis-associated cases, and malignancy-associated cases indicate that pooling all cases into a single disease category would risk obscuring biologically and clinically important differences. Future studies should avoid pooling these cases unless antigen status, associated conditions, crescent burden, treatment timing, and follow-up outcomes are reported consistently.

Accordingly, the current literature supports descriptive synthesis and phenotype mapping, but not robust comparative inference across the full overlap spectrum. Descriptive comparisons of PLA2R-positive versus PLA2R-negative cases, RPGN presentation, regional concentration in Asia and North America, rituximab use, and ANCA-related features are feasible, but formal prognosis or treatment-effect comparisons remain limited by selective antigen testing, sparse longitudinal ANCA measurements, inconsistent remission definitions, variable assessment time points, and incomplete follow-up. These constraints also limit the feasibility of a broad systematic review of pooled treatment effects or prognosis at present.

A more feasible next step is phenotype-specific comparative research separating likely MPO-associated / ANCA-GN-related MN from primary-MN-like and secondary / associated forms. As summarized in Online Resource 4, future studies should refine and validate explicit phenotype criteria and report the minimum data elements needed for meaningful comparison, including standardized antigen testing, percent crescent data, longitudinal ANCA assessment, standardized outcome definitions, assessment time points, and explicit follow-up.

This review has several limitations. The search was limited to PubMed, Web of Science, and CENTRAL with reference-list screening, so studies indexed elsewhere or reported with atypical terminology may have been missed. The literature was dominated by case reports and small case series, with inconsistent antigen testing, outcome definitions, assessment time points, and follow-up. Because this was a scoping review, we did not perform a formal critical appraisal or risk-of-bias assessment of individual studies; therefore, it maps the extent and characteristics of the literature but cannot determine certainty of evidence or support causal or treatment-effect conclusions.

In conclusion, coexisting ANCA-GN and MN appears to represent a rare but clinically important spectrum of overlapping phenotypes. The literature supports a phenotype-oriented framework and highlights situations in which the overlap should be suspected, but it does not justify treating all reported cases as a single disease entity for comparative inference. More informative synthesis will require standardized phenotype definitions, broader antigen profiling, longitudinal serologic assessment, consistent outcome definitions, and explicit follow-up reporting. International multicenter registries and harmonized reporting standards are likely to be important for future evidence synthesis.

Abbreviations: PRISMA-ScR, Preferred Reporting Items for Systematic reviews and Meta-Analyses extension for Scoping Reviews.

## Supplementary Information

Below is the link to the electronic supplementary material.

Supplementary Material 1

Supplementary Material 2

Supplementary Material 3

Supplementary Material 4

Supplementary Material 5

Supplementary Material 6

Supplementary Material 7
