# Supplementary material for: Coexistence of ANCA-associated glomerulonephritis and membranous nephropathy: a scoping review
Source: Clin Exp Nephrol. 2026 Jun 10;30(10):1451–6. doi: 10.1007/s10157-026-02904-y (PMC13582339; doi:10.1007/s10157-026-02904-y)
Supplement: Supplementary file 1 [file 10157_2026_2904_MOESM1_ESM.pdf]

Online Resource 1. Search Strategy

PubMed Search Strategy:

#1 "Glomerulonephritis, Membranous"[MeSH]

#2 "Membranous Nephropathy"[Title/Abstract]

#3 "Membranous Glomerulopathy"[Title/Abstract]

#4 "Membranous Glomerulonephritis"[Title/Abstract]

#5 "Epimembranous Nephropathy"[Title/Abstract]

#6 "Anti-Neutrophil Cytoplasmic Antibody-Associated Vasculitis"[MeSH]

#7 "ANCA-Associated Vasculitis"[Title/Abstract]

#8 "ANCA-associated glomerulonephritis"[Title/Abstract]

#9 "ANCA associated glomerulonephritis"[Title/Abstract]

#10 "Microscopic Polyangiitis"[Title/Abstract]

#11 "Granulomatosis with Polyangiitis"[Title/Abstract]

#12 "Wegener*"[Title/Abstract]

#13 "Churg-Strauss"[Title/Abstract]

#14 "Eosinophilic Granulomatosis with Polyangiitis"[Title/Abstract]

#15 "Anti-neutrophil cytoplasmic antibod*"[Title/Abstract]

#16 "ANCA"[Title/Abstract]

#17 #1 OR #2 OR #3 OR #4 OR #5

#18 #6 OR #7 OR #8 OR #9 OR #10 OR #11 OR #12 OR #13 OR #14 OR #15 OR #16

#19 #17 AND #18

Web of Science search strategy:

#1 TS= ("Membranous Nephropathy")

#2 TS= ("Membranous Glomerulopathy")

#3 TS= ("Membranous Glomerulonephritis")

#4 TS= ("ANCA-Associated Vasculitis")

#5 TS= ("ANCA associated glomerulonephritis")

#6 TS= ("Microscopic Polyangiitis")

#7 TS= ("Granulomatosis with Polyangiitis")

#8 TS= ("Wegener*")

#9 TS= (“Churg-Strauss")

#10 TS= ("Eosinophilic Granulomatosis with Polyangiitis")

#11 TS= ("Anti-neutrophil cytoplasmic antibod*")

#12 TS= ("ANCA")

#13 #1 OR #2 OR #3

#14 #4 OR #5 OR #6 OR #7 OR #8 OR #9 OR #10 OR #11 OR #12

#15 #13 AND #14

CENTRAL search strategy:

#1 MeSH descriptor: [Glomerulonephritis, Membranous] explode all trees

#2 ("Membranous Nephropathy" OR "Membranous Glomerulopathy" OR "Membranous Glomerulonephritis"):ti,ab,kw

#3 #1 OR #2

#4 MeSH descriptor: [Anti-Neutrophil Cytoplasmic Antibody-Associated Vasculitis] explode all trees

#5 ("ANCA-Associated Vasculitis" OR "ANCA associated glomerulonephritis" OR "Microscopic Polyangiitis" OR "Granulomatosis with Polyangiitis" OR "Wegener*" OR "Churg-Strauss" OR "Eosinophilic Granulomatosis with Polyangiitis" OR "Anti-neutrophil cytoplasmic antibod*" OR "ANCA"):ti,ab,kw

#6 #4 OR #5

#7 #3 AND #6

Title: Coexistence of ANCA-Associated Glomerulonephritis and Membranous Nephropathy: A Scoping Review

Journal name: Clinical and Experimental Nephrology

Author names: Ryosuke Saiki, Kan Katayama, Mutsuki Mori, Kaoru Dohi

Affiliation: Department of Cardiology and Nephrology, Mie University Graduate School of Medicine

E-mail: ryosuke-s@med.mie-u.ac.jp
