# Supplementary material for: Coexistence of ANCA-associated glomerulonephritis and membranous nephropathy: a scoping review
Source: Clin Exp Nephrol. 2026 Jun 10;30(10):1451–6. doi: 10.1007/s10157-026-02904-y (PMC13582339; doi:10.1007/s10157-026-02904-y)
Supplement: Supplementary file 2 [file 10157_2026_2904_MOESM2_ESM.pdf]

Online Resource 2. Proposed phenotypic categories of ANCA-associated glomerulonephritis with membranous nephropathy and operational criteria used in this review

Phenotypic Category	Working Definition Used in This Review	Key Supportive Findings	Role of Temporal Relationship
Likely ANCA-GN-Related MN	Cases with ANCA-GN-related clinicopathologic features and no antigen-defined primary-MN-like phenotype or clear secondary/associated cause.	MPO- or PR3-ANCA positivity, frequent crescents, and a small subset with explicit MPO deposition or MPO-associated wording.	Concurrent onset may support assignment, but temporal relationship alone is insufficient.
Likely Primary-MN-Like Overlap With ANCA-GN	Cases with serum and/or kidney PLA2R positivity and/or THSD7A positivity.	PLA2R and/or THSD7A positivity, with or without IgG4-dominant features.	MN preceding ANCA-GN may support coincidental coexistence, but concurrent onset does not exclude this category.
Secondary / Associated MN	Cases with a clear drug trigger, lupus/class V lupus nephritis, rheumatoid arthritis, or an explicit malignancy/neoplasm-associated presentation.	PTU/MMI/hydralazine/cocaine-levamisole, gold, G-CSF, rheumatoid arthritis-associated, lupus/class V LN, and carcinoma/neoplasm-associated presentations.	Suggestive when drug exposure or the associated disease predates kidney findings, but temporal relationship alone is insufficient.
Unclassifiable	ANCA-seropositive cases lacking sufficient evidence for confident assignment to the other phenotypic categories.	ANCA seropositivity documented, but atypical profile or insufficient antigen/pathology data.	Temporal relationship may be described when available, but it does not determine assignment.

- Temporal relationship was defined as concurrent onset (≤6 months between renal biopsy and first positive ANCA test), MN preceding (>6 months), or ANCA preceding (>6 months).
- Cases were assigned hierarchically: antigen-defined primary-MN-like overlap was prioritized when PLA2R and/or THSD7A positivity was present; secondary / associated MN was assigned for clear drug-triggered, lupus/class V lupus nephritis, explicit malignancy/neoplasm-associated, or rheumatoid arthritis-associated presentations without antigen-defined primary-MN-like features; likely ANCA-GN-related MN was assigned when neither applied; otherwise, cases were considered unclassifiable.
- Temporal relationship was used as supportive information and not as the sole basis for classification.

Title: Coexistence of ANCA-Associated Glomerulonephritis and Membranous Nephropathy: A Scoping Review Journal name: Clinical and Experimental Nephrology Author names: Ryosuke Saiki, Kan Katayama, Mutsuki Mori, Kaoru Dohi Affiliation: Department of Cardiology and Nephrology, Mie University Graduate School of Medicine E-mail: ryosuke-s@med.mie-u.ac.jp
