# Supplementary material for: Coexistence of ANCA-associated glomerulonephritis and membranous nephropathy: a scoping review
Source: Clin Exp Nephrol. 2026 Jun 10;30(10):1451–6. doi: 10.1007/s10157-026-02904-y (PMC13582339; doi:10.1007/s10157-026-02904-y)
Supplement: Supplementary file 3 [file 10157_2026_2904_MOESM3_ESM.pdf]

Online Resource 3. Clinicopathologic Features, Treatment Patterns, and Kidney Outcomes Across Phenotypic Categories

Characteristic	Overall Evaluable Cases	Likely ANCA-GN-Related MN	Likely Primary-MN-Like Overlap With ANCA-GN	Secondary / Associated MN	Unclassifiable
**Evidence base**					
Studies/patients, n	69 studies / 113 patients	35 studies / 66 patients	7 studies / 10 patients	26 studies / 31 patients	5 studies / 6 patients
**Demographics**					
Age at diagnosis, median (range), y	60 (10-88)	63 (10-88)	57 (41-79)	52 (21-81)	64.5 (25-79)
Female sex, n/N (%)	46/113 (40.7%)	26/66 (39.4%)	3/10 (30.0%)	16/31 (51.6%)	1/6 (16.7%)
Region: Asia, n/N (%)	49/113 (43.4%)	27/66 (40.9%)	6/10 (60.0%)	14/31 (45.2%)	2/6 (33.3%)
Region: North America, n/N (%)	41/113 (36.3%)	20/66 (30.3%)	4/10 (40.0%)	13/31 (41.9%)	4/6 (66.7%)
Region: Europe, n/N (%)	20/113 (17.7%)	16/66 (24.2%)	0/10 (0.0%)	4/31 (12.9%)	0/6 (0.0%)
Region: Other, n/N (%)	3/113 (2.7%)	3/66 (4.5%)	0/10 (0.0%)	0/31 (0.0%)	0/6 (0.0%)
**Clinical presentation**					
RPGN presentation, n/N (%)	75/88 (85.2%)	42/50 (84.0%)	6/7 (85.7%)	22/26 (84.6%)	5/5 (100.0%)
Serum creatinine at diagnosis, median (range), mg/dL	2.75 (0.13-14.5)	3 (0.13-14.5)	2.75 (0.73-13.4)	2.2 (0.43-9.2)	2.91 (1.13-8.9)
Proteinuria at diagnosis, median (range), g/day or g/gCr	3.7 (0.2-37.8)	4.96 (0.31-37.8)	6.74 (1.51-15.9)	2 (0.2-10.6)	2.67 (1-4.35)

Characteristic	Overall Evaluable Cases	Likely ANCA-GN-Related MN	Likely Primary-MN-Like Overlap With ANCA-GN	Secondary / Associated MN	Unclassifiable
Hematuria reported, n/N (%)	81/83 (97.6%)	43/44 (97.7%)	9/9 (100.0%)	26/27 (96.3%)	3/3 (100.0%)
Kidney-limited disease, n/N (%)	54/95 (56.8%)	31/54 (57.4%)	5/7 (71.4%)	16/31 (51.6%)	2/3 (66.7%)
Any extrarenal manifestation, n/N (%)	41/95 (43.2%)	23/54 (42.6%)	2/7 (28.6%)	15/31 (48.4%)	1/3 (33.3%)
Pulmonary involvement, n/N (%)	29/95 (30.5%)	20/54 (37.0%)	2/7 (28.6%)	6/31 (19.4%)	1/3 (33.3%)
ENT involvement, n/N (%)	11/95 (11.6%)	8/54 (14.8%)	1/7 (14.3%)	2/31 (6.5%)	0/3 (0.0%)
Skin involvement, n/N (%)	13/95 (13.7%)	3/54 (5.6%)	1/7 (14.3%)	8/31 (25.8%)	1/3 (33.3%)
**Serologic and antigen findings**					
MPO-ANCA positive, n/N (%)	75/112 (67.0%)	43/66 (65.2%)	6/10 (60.0%)	25/31 (80.6%)	1/5 (20.0%)
PR3-ANCA positive, n/N (%)	19/112 (17.0%)	8/66 (12.1%)	2/10 (20.0%)	9/31 (29.0%)	0/5 (0.0%)
Double-positive ANCA, n/N (%)	9/112 (8.0%)	1/66 (1.5%)	0/10 (0.0%)	8/31 (25.8%)	0/5 (0.0%)
Noncanonical or unspecified ANCA subtype, n/N (%)	27/112 (24.1%)	16/66 (24.2%)	2/10 (20.0%)	5/31 (16.1%)	4/5 (80.0%)
PLA2R assessed, n/N (%)	31/113 (27.4%)	13/66 (19.7%)	10/10 (100.0%)	7/31 (22.6%)	1/6 (16.7%)
PLA2R positive, n/N (%)	10/31 (32.3%)	0/13 (0.0%)	10/10 (100.0%)	0/7 (0.0%)	0/1 (0.0%)
THSD7A assessed, n/N (%)	1/113 (0.9%)	1/66 (1.5%)	0/10 (0.0%)	0/31 (0.0%)	0/6 (0.0%)
THSD7A positive, n/N (%)	0/1 (0.0%)	0/1 (0.0%)	NR	NR	NR
IgG subclass assessed, n/N (%)	19/113 (16.8%)	9/66 (13.6%)	5/10 (50.0%)	5/31 (16.1%)	0/6 (0.0%)

Characteristic	Overall Evaluable Cases	Likely ANCA-GN-Related MN	Likely Primary-MN-Like Overlap With ANCA-GN	Secondary / Associated MN	Unclassifiable
IgG4-dominant or IgG4-positive pattern, n/N (%)	11/19 (57.9%)	6/9 (66.7%)	4/5 (80.0%)	1/5 (20.0%)	NR
Non-IgG4-dominant or mixed pattern, n/N (%)	8/19 (42.1%)	3/9 (33.3%)	1/5 (20.0%)	4/5 (80.0%)	NR
**Pathologic findings**					
Crescents present, n/N (%)	97/109 (89.0%)	60/64 (93.8%)	8/9 (88.9%)	23/30 (76.7%)	6/6 (100.0%)
Glomeruli with crescents, median (range), %	33 (4-92)	33 (4-92)	31 (7-58)	33.5 (4-73)	35 (8-70)
**Associated conditions and triggers**					
Drug exposure implicated, n/N (%)	20/100 (20.0%)	0/61 (0.0%)	0/6 (0.0%)	20/31 (64.5%)	0/2 (0.0%)
Coexisting autoimmune disease, n/N (%)	13/98 (13.3%)	0/61 (0.0%)	1/7 (14.3%)	12/28 (42.9%)	0/2 (0.0%)
Coexisting malignancy, n/N (%)	4/99 (4.0%)	0/61 (0.0%)	0/7 (0.0%)	4/29 (13.8%)	0/2 (0.0%)
Concurrent onset (≤6 months), n/N (%)	101/113 (89.4%)	60/66 (90.9%)	7/10 (70.0%)	29/31 (93.5%)	5/6 (83.3%)
MN preceding ANCA-GN (>6 months), n/N (%)	7/113 (6.2%)	4/66 (6.1%)	1/10 (10.0%)	2/31 (6.5%)	0/6 (0.0%)
ANCA preceding MN (>6 months), n/N (%)	5/113 (4.4%)	2/66 (3.0%)	2/10 (20.0%)	0/31 (0.0%)	1/6 (16.7%)
**Induction treatment**					
Glucocorticoids, n/N (%)	95/100 (95.0%)	60/61 (98.4%)	5/6 (83.3%)	29/31 (93.5%)	1/2 (50.0%)
Cyclophosphamide, n/N (%)	58/100 (58.0%)	38/61 (62.3%)	5/6 (83.3%)	15/31 (48.4%)	0/2 (0.0%)
Rituximab, n/N (%)	10/100 (10.0%)	6/61 (9.8%)	1/6 (16.7%)	3/31 (9.7%)	0/2 (0.0%)
Plasma exchange, n/N (%)	13/100 (13.0%)	6/61 (9.8%)	1/6 (16.7%)	6/31 (19.4%)	0/2 (0.0%)

Characteristic	Overall Evaluable Cases	Likely ANCA-GN-Related MN	Likely Primary-MN-Like Overlap With ANCA-GN	Secondary / Associated MN	Unclassifiable
Other immunosuppression, n/N (%)	27/100 (27.0%)	17/61 (27.9%)	1/6 (16.7%)	9/31 (29.0%)	0/2 (0.0%)
**Outcomes**					
ESKD, n/N (%)	24/97 (24.7%)	17/60 (28.3%)	1/6 (16.7%)	5/27 (18.5%)	1/4 (25.0%)

- This online resource is based on the 113 phenotype-evaluable individual-level cases. The aggregate-only cohort study [75] was not included because it did not provide details.
- Denominators vary by variable and include only cases with reported results; cases listed as not reported were excluded, whereas explicitly negative cases were included.
- Concurrent onset was defined as ≤6 months between renal biopsy and first positive ANCA test.

Title: Coexistence of ANCA-Associated Glomerulonephritis and Membranous Nephropathy: A Scoping Review Journal name: Clinical and Experimental Nephrology Author names: Ryosuke Saiki, Kan Katayama, Mutsuki Mori, Kaoru Dohi Affiliation: Department of Cardiology and Nephrology, Mie University Graduate School of Medicine E-mail: ryosuke-s@med.mie-u.ac.jp
