# Supplementary material for: Coexistence of ANCA-associated glomerulonephritis and membranous nephropathy: a scoping review
Source: Clin Exp Nephrol. 2026 Jun 10;30(10):1451–6. doi: 10.1007/s10157-026-02904-y (PMC13582339; doi:10.1007/s10157-026-02904-y)
Supplement: Supplementary file 4 [file 10157_2026_2904_MOESM4_ESM.pdf]

Online Resource 4. Reporting gaps and data elements needed for future comparative studies

Domain	Data Element for Comparison	Current Availability in This Review	Recommended Standard for Future Studies
Phenotype definition	Prespecified phenotype assignment rule	Phenotype framework: 0/70	Develop and validate explicit phenotype assignment criteria, building on operational frameworks such as the one proposed in this review.
ANCA serology and longitudinal assessment	Baseline ANCA type/titer and follow-up ANCA status	Baseline ANCA type/titer reported: 55/70; follow-up ANCA available: 18/70	Report assay method, target antigen (MPO/PR3), units, cutoff, and ANCA follow-up with timing; provide serial values when available.
MN antigen profiling	PLA2R and THSD7A status	PLA2R/THSD7A status reported: 23/70; THSD7A rarely assessed	Report serum and kidney PLA2R/THSD7A separately, and state when not tested.
Pathology	Percent crescents	Percent crescents reported: 50/70	Report total glomeruli and percent crescents.
Outcome definition, assessment timing, and follow-up duration	Remission definition, assessment time points, and follow-up duration	Remission definition: 3/70; assessment time points and follow-up duration were insufficiently reported.	Use standardized outcome definitions and report prespecified assessment time points and total follow-up duration.

- Availability was assessed at the study level, using the 70 included studies as the denominator unless otherwise specified. Timing remained classifiable in all 69 individual-level studies contributing phenotype-evaluable cases; the aggregate-only cohort was not applicable to that item.
- Assessment time points were defined as explicit post-treatment or outcome-evaluation time points. Follow-up duration was defined as the total reported observation interval or last reported follow-up duration for at least one included case or cohort.

Title: Coexistence of ANCA-Associated Glomerulonephritis and Membranous Nephropathy: A Scoping Review Journal name: Clinical and Experimental Nephrology Author names: Ryosuke Saiki, Kan Katayama, Mutsuki Mori, Kaoru Dohi Affiliation: Department of Cardiology and Nephrology, Mie University Graduate School of Medicine E-mail: ryosuke-s@med.mie-u.ac.jp
