# Supplementary material for: Coexistence of ANCA-associated glomerulonephritis and membranous nephropathy: a scoping review
Source: Clin Exp Nephrol. 2026 Jun 10;30(10):1451–6. doi: 10.1007/s10157-026-02904-y (PMC13582339; doi:10.1007/s10157-026-02904-y)
Supplement: Supplementary file 5 [file 10157_2026_2904_MOESM5_ESM.pdf]

Online Resource 6. Aggregate-only cohort characteristics of the included study with coexisting ANCA-associated glomerulonephritis and membranous nephropathy

Author	Year	Country	N	Age	Female	Malignancy	Autoimmune diseases other than ANCA	Cre	PU	Hematuria	ANCA	PLA2R serum	PLA2R kidney	THSD7A	IgG subclass	Crescent	Extrarenal lesions	Treatment	Outcome
Zou R [75]	2015	China	27	52.4 ± 17.7	10 (37.0)	Excluded per eligibility criteria	Excluded per eligibility criteria	6.41 ± 5.15	3.84 ± 2.35	Gross hematuria 33%	MPO-ANCA 92.6%; PR3-ANCA 7.4%	12.5%	6.6%	NR	IgG1 46.7%; IgG2 53.3%; IgG3 66.7%; IgG4 20.0%	54.9 ± 34.3%	L 51.9%; ENT 55.5%; gastrointestinal 22.2%	Steroids 92.7%; CPA 92.7%; PE 37.0%	ESKD 48.1%

This supplementary table summarizes the aggregate-only cohort study that was not merged into the phenotype-specific columns in the main manuscript.

Age, Cre, PU, and Crescent are presented as cohort-level summary values reported in the original article (mean ± SD). Hematuria reporting reflects the original study definition, which in this cohort was gross hematuria. PLA2R kidney refers to tissue staining. Malignancy and Autoimmune diseases other than ANCA are listed as Excluded per eligibility criteria because the original cohort excluded such patients by study design.

Abbreviations: ANCA; antineutrophil cytoplasmic antibody, CPA; Cyclophosphamide, Cre; Creatinine, ENT; Ear and/or nose and/or throat, ESKD; End-stage kidney disease, L; Lungs, NR; Not reported, PE; Plasma exchange, PLA2R; phospholipase A2 receptor, PR3; Proteinase 3, PU; Proteinuria, THSD7A; thrombospondin type-1 domain-containing 7A.

Title: Coexistence of ANCA-Associated Glomerulonephritis and Membranous Nephropathy: A Scoping Review Journal name: Clinical and Experimental Nephrology Author names: Ryosuke Saiki, Kan Katayama, Mutsuki Mori, Kaoru Dohi Affiliation: Department of Cardiology and Nephrology, Mie University Graduate School of Medicine E-mail: ryosuke-s@med.mie-u.ac.jp
