# Supplementary material for: Coexistence of ANCA-associated glomerulonephritis and membranous nephropathy: a scoping review
Source: Clin Exp Nephrol. 2026 Jun 10;30(10):1451–6. doi: 10.1007/s10157-026-02904-y (PMC13582339; doi:10.1007/s10157-026-02904-y)
Supplement: Supplementary file 6 [file 10157_2026_2904_MOESM6_ESM.pdf]

Online Resource 7. Descriptive comparison of PLA2R-positive and PLA2R-negative cases

Characteristic	Overall PLA2R-assessed cases	PLA2R-positive	PLA2R-negative
**Evidence base**			
Studies/patients, n	22 studies / 31 patients	7 studies / 10 patients	16 studies / 21 patients
**Demographics**			
Age at diagnosis, median (range), y	56 (19-88)	57 (41-79)	55 (19-88)
Female sex, n/N (%)	12/31 (38.7%)	3/10 (30.0%)	9/21 (42.9%)
Region: Asia, n/N (%)	13/31 (41.9%)	6/10 (60.0%)	7/21 (33.3%)
Region: North America, n/N (%)	16/31 (51.6%)	4/10 (40.0%)	12/21 (57.1%)
Region: Europe, n/N (%)	1/31 (3.2%)	0/10 (0.0%)	1/21 (4.8%)
Region: Other, n/N (%)	1/31 (3.2%)	0/10 (0.0%)	1/21 (4.8%)
**Clinical presentation**			
RPGN presentation, n/N (%)	18/22 (81.8%)	6/7 (85.7%)	12/15 (80.0%)
Serum creatinine at diagnosis, median (range), mg/dL	2.61 (0.73-13.4)	2.75 (0.73-13.4)	2.61 (0.86-9.2)
Proteinuria at diagnosis, median (range), g/day or g/gCr	3 (0.2-19.2)	6.74 (1.51-15.9)	2.57 (0.2-19.2)
Hematuria reported, n/N (%)	29/29 (100.0%)	9/9 (100.0%)	20/20 (100.0%)
Kidney-limited disease, n/N (%)	9/22 (40.9%)	5/7 (71.4%)	4/15 (26.7%)
Any extrarenal manifestation, n/N (%)	13/22 (59.1%)	2/7 (28.6%)	11/15 (73.3%)
Pulmonary involvement, n/N (%)	9/22 (40.9%)	2/7 (28.6%)	7/15 (46.7%)
ENT involvement, n/N (%)	6/22 (27.3%)	1/7 (14.3%)	5/15 (33.3%)
Skin involvement, n/N (%)	4/22 (18.2%)	1/7 (14.3%)	3/15 (20.0%)
**Serologic and antigen findings**			
MPO-ANCA positive, n/N (%)	22/31 (71.0%)	6/10 (60.0%)	16/21 (76.2%)
PR3-ANCA positive, n/N (%)	4/31 (12.9%)	2/10 (20.0%)	2/21 (9.5%)
Double-positive ANCA, n/N (%)	1/31 (3.2%)	0/10 (0.0%)	1/21 (4.8%)
IgG subclass assessed, n/N (%)	12/31 (38.7%)	5/10 (50.0%)	7/21 (33.3%)
IgG4-dominant or IgG4-positive pattern, n/N (%)	6/12 (50.0%)	4/5 (80.0%)	2/7 (28.6%)
**Pathologic findings**			
Crescents present, n/N (%)	27/30 (90.0%)	8/9 (88.9%)	19/21 (90.5%)
Glomeruli with crescents, median (range), %	21 (0-80)	25 (0-58)	20 (0-80)
**Associated conditions and triggers**			
Drug exposure implicated, n/N (%)	4/22 (18.2%)	0/7 (0.0%)	4/15 (26.7%)
Coexisting autoimmune disease, n/N (%)	6/22 (27.3%)	2/7 (28.6%)	4/15 (26.7%)
Concurrent onset (≤6 months), n/N (%)	26/31 (83.9%)	7/10 (70.0%)	19/21 (90.5%)
MN preceding ANCA-GN (>6 months), n/N (%)	2/31 (6.5%)	1/10 (10.0%)	1/21 (4.8%)
ANCA preceding MN (>6 months), n/N (%)	3/31 (9.7%)	2/10 (20.0%)	1/21 (4.8%)
**Induction treatment**			

Characteristic	Overall PLA2R-assessed cases	PLA2R-positive	PLA2R-negative
Glucocorticoids, n/N (%)	20/22 (90.9%)	5/6 (83.3%)	15/16 (93.8%)
Cyclophosphamide, n/N (%)	12/22 (54.5%)	5/6 (83.3%)	7/16 (43.8%)
Rituximab, n/N (%)	6/22 (27.3%)	1/6 (16.7%)	5/16 (31.2%)
Plasma exchange, n/N (%)	5/22 (22.7%)	1/6 (16.7%)	4/16 (25.0%)
Other immunosuppression, n/N (%)	6/22 (27.3%)	2/6 (33.3%)	4/16 (25.0%)
**Outcomes**			
ESKD, n/N (%)	5/22 (22.7%)	1/6 (16.7%)	4/16 (25.0%)

`PLA2R-positive` was defined as serum and/or kidney PLA2R positivity. Cases with `NR`, `Not tested`, or otherwise unassessable PLA2R status were excluded from the comparison columns.

Marker positivity rows use tested cases as the denominator. Clinical presentation, extrarenal manifestation, associated-condition, treatment, and outcome rows use cases with reported data for that item. Timing rows use all 31 PLA2R-assessed cases. A study may contribute patients to both `PLA2R-positive` and `PLA2R-negative` columns; therefore, study counts do not sum across subgroup columns. Testing was selective, and the table should be interpreted as descriptive and exploratory. No formal statistical comparison was performed.

Abbreviations: ANCA; antineutrophil cytoplasmic antibody, ENT; Ear and/or nose and/or throat, ESKD; End-stage kidney disease, MPO; Myeloperoxidase, NR; Not reported, PLA2R; phospholipase A2 receptor, PR3; Proteinase 3, RPGN; rapidly progressive glomerulonephritis, THSD7A; thrombospondin type-1 domain-containing 7A.

Title: Coexistence of ANCA-Associated Glomerulonephritis and Membranous Nephropathy: A Scoping Review Journal name: Clinical and Experimental Nephrology Author names: Ryosuke Saiki, Kan Katayama, Mutsuki Mori, Kaoru Dohi Affiliation: Department of Cardiology and Nephrology, Mie University Graduate School of Medicine E-mail: ryosuke-s@med.mie-u.ac.jp
