# Supplementary material for: Coexistence of ANCA-associated glomerulonephritis and membranous nephropathy: a scoping review
Source: Clin Exp Nephrol. 2026 Jun 10;30(10):1451–6. doi: 10.1007/s10157-026-02904-y (PMC13582339; doi:10.1007/s10157-026-02904-y)
Supplement: Supplementary file 7 [file 10157_2026_2904_MOESM7_ESM.pdf]

Online Resource 5. Individual-level characteristics of included cases with coexisting ANCA-associated glomerulonephritis and membranous nephropathy

Author	Year	Country	Age	Sex	Comorbidity	Malignancy	Autoimmune diseases other than ANCA(0/1)	Cre	PU	RPGN(0/1)	Hematuria	ANCA	PLA2R serum	PLA2R kidney	TSHD7A	IgG subclass	Crescent	Extrarenal lesions	Treatment	Outcome Cre	Outcome UP	Remission	Timing	Trigger/etiology
Nakada Y [13]	2015	Japan	70	M	Sick sinus syndrome, interstitial pneumonia, angina pectoris	0	0	3.2	5.12	1	1	p-ANCA; MPO/PR3 negative	NR	NR	NR	NR	0.92	E, L	PO steroids	RRT	NR	NR	Concurrent	
Kim YH [14]	2015	Korea	65	M	HT	0	0	9.7	5.62	1	1	p-ANCA 1:8 antibody index	NR	NR	NR	NR	0.474	ENT, L	IV/PO steroids, PO CPA	RRT	NR	NR	Concurrent	
Matsuda J [15]	2013	Japan	71	M	COPD	0	0	2.7	4.35	1	1	Elastase 11.5 (>4.0, positive); BPI 5.3 (>4.0, positive)	NR	NR	NR	NR	0.7	None	IV/PO steroids	1.5	<0.5	NR	Concurrent	
Hu Z [16]	2014	China	51	F	HT	0	0	1.4	11	1	1	MPO 62 IU/ml	+ (16.4 μg/ml)	+	NR	IgG4 dominant	0.58	None	IV/PO steroids, PO CPA	1.36	4.29	NR	Concurrent	
Mahmood SB [17]	2019	USA	63	F	HCV	0	0	NR	8.04	0	1	MPO	-	NR	NR	IgG4 normal (not elevated)	0	ENT, L, N	PO steroids, RTX, mepolizumab, omalizumab	NR	0.95	NR	Concurrent	
Fujii T [18]	2003	Japan	64	M	HTH	0	0	1.75	1.4	1	1	MPO 74 EU; PR3 33 EU	NR	NR	NR	NR	0.44	None	IV/PO steroids, change PTU to MMI	1.2	0.7	NR	Concurrent	PTU-induced
Shimada M [19]	2013	Japan	73	M	HT, GU, Vasospastic angina, Colon cancer	1	0	5.4	2.3	1	1	MPO >640 EU	NR	NR	NR	IgG4 not dominant	0.73	None	IV/PO steroids	RRT	NA	NR	Concurrent	
Uyama S [20]	2013	Japan	72	F	None	0	0	4.05	2.28	1	1	MPO 38 IU/ml	NR	NR	NR	IgG4 negative	0.35	None	IV/PO steroids	2.0-3.0	around 1	NR	Concurrent	
Takahashi N [21]	2009	Japan	56	M	None	0	0	2.6	5.6	1	1	MPO 640 IU/ml	NR	NR	NR	NR	0.46	L, N	IV/PO steroids, CyA	Death	Death	NR	Concurrent	
Collister D [22]	2017	Canada	53	M	HT, DLP, Lumbar surgery, Chronic pain syndrome	0	0	1.7	0.2	1	1	MPO 167 RU/mL; PR3 8 RU/mL	NR	-	NR	NR	0.12	Skin	IV/PO steroids	1.69	0.16	NR	Concurrent	Cocaine use
Collister D [22]	2017	Canada	35	M	None	0	0	1.7	0.59	1	1	MPO >200 RU/mL; PR3 24 RU/mL	NR	NR	NR	NR	0.13	ENT, L	PO steroids, AZA, IV CPA	1.37	0.06	NR	Concurrent	Cocaine use
Collister D [22]	2017	Canada	34	M	HT, Severe obesity	0	0	0.8	NR	0	1	MPO 91 RU/mL; PR3 74 RU/mL	NR	NR	NR	NR	0	Skin	PO steroids, AZA, PO CPA, TAC	0.64	0.15	NR	Concurrent	Cocaine use
Sekine S [23]	2002	Japan	81	F	RA	0	1(RA)	1.25	5.95	0	1	MPO 166 EU	NR	NR	NR	NR	0	L	PO steroids	1.5	0.5	NR	Concurrent	
Meisels IS [24]	1992	USA	50	M	RA, Idiopathic thrombocytopenic purpura	0	1(RA)	3.7	>3.5	1	1	MPO; PR3	NR	NR	NR	NR	0.5	None	IV/PO steroids, PE	0.8	Negative	NR	Concurrent	
Etter C [25]	2011	Switzerland	52	F	NR	0	0	4.84	1.7	1	1	MPO 1700 EU	NR	NR	NR	NR	0.556	L	IV/PO steroids, AZA, PE	1.54	0.23	NR	Concurrent	
Kakita T [26]	2013	Japan	21	F	HTH, ID, Congenital diaphragmatic hernia, Hypoxic ischemic encephalopathy	0	0	0.43	6.4	0	1	MPO 79 EU	NR	NR	NR	NR	0.34	None	IV/PO steroids, AZA, IV CPA, CyA stop MMI	NA	NR	NR	Concurrent	MMI-induced
Tominaga K [27]	2018	Japan	52	M	None	0	0	1.99	15.9	1	1	MPO 19.4 EU	-	+	NR	IgG4 positive	0.46	L	NR	NR	NR	NR	ANCA preceding	
Tominaga K [27]	2018	Japan	63	F	Relapsing polychondritis	0	1(Relapsing polychondritis)	0.73	1.6	1	1	MPO 25.6 EU	-	+	NR	IgG4 negative	0.11	None	NR	NR	NR	NR	ANCA preceding	
Jiang S [28]	2022	China	25	M	NA	NR	NR	1.13	NR	1	1	NR	NR	NR	NR	NR	0.3	L, Skin	NA	NA	NA	NR	Concurrent	
Zuo Y [29]	2021	USA	61	F	NA	NR	NR	8	8	NR	1	c-ANCA; MPO	NR	-	NR	NR	0.22	NA	NA	NA	NA	NR	Concurrent	
Zuo Y [29]	2021	USA	63	F	NA	NR	NR	4.1	NA	NR	1	c-ANCA	NR	-	NR	NR	0.2	NA	NA	NA	NA	NR	Concurrent	
Zuo Y [29]	2021	USA	47	M	NA	NR	NR	2.1	0.31	NR	1	p-ANCA 1:160; MPO 58.5	NR	-	NR	NR	0.17	NA	NA	NA	NA	NR	Concurrent	
Zuo Y [29]	2021	USA	81	M	NA	NR	NR	3.7	4.98	NR	1	p-ANCA	NR	-	NR	NR	0.56	NA	NA	NA	NA	NR	Concurrent	
Zuo Y [29]	2021	USA	54	M	NA	NR	NR	1.8	1	NR	1	type unknown	NR	-	NR	NR	0.08	NA	NA	NA	NA	NR	ANCA preceding	
Zuo Y [29]	2021	USA	58	M	NA	NR	NR	3.5	NA	NR	1	p-ANCA	NR	+	NR	NR	0.25	NA	IV CPA, AZA	around 2.0	NA	NR	Concurrent	
Zuo Y [29]	2021	USA	79	F	NA	NR	NR	3.7	4.5	NR	1	PR3	NR	+	NR	NR	0.31	NA	NA	NA	NA	NR	Concurrent	
Zuo Y [29]	2021	USA	88	M	MGUS	NR	NR	4.8	NA	NR	NA	p-ANCA	NR	-	NR	NR	0.2	NA	PO steroids, PO CPA	2.7	NA	NR	Concurrent	
Zuo Y [29]	2021	USA	60	M	NA	NR	NR	6.6	NA	NR	NA	type unknown	NR	+	NR	NR	0.07	NA	NA	NA	NA	NR	Concurrent	
Watanabe S [30]	2007	Japan	79	F	None	0	0	0.6	5.3	0	1	MPO 425 EU	NR	NR	NR	IgG4 dominant	0.09	None	IV/PO steroids	0-1.0(stable)	Negative	NR	Concurrent	
P'ng CH [31]	2017	Australia	55	M	ILD, T2DM, HT,DLP, BPH	0	0	7.61	2.57	1	1	MPO >100 U/mL	-	NR	NR	NR	0.63	L	IV/PO steroids, CPA, PE	3.71	NA	NR	Concurrent	
Fang W [32]	2025	China	50	F	HT, hypothyroidism	0	0	0.87	1.59	0	1	p-ANCA; MPO	NR	-	NR	NR	0.36	ENT, L	IV/PO steroids, CPA, MMF	0.71	0.03	NR	Concurrent	
Tse WY [33]	1997	UK	30	M	None	0	0	1.36	1.5	NR	1	c-ANCA 1:100	NR	NR	NR	NR	0.5	Skin	steroids, CPA	1.35	NA	NR	Concurrent	
Tse WY [33]	1997	UK	64	M	T2DM	0	0	5.76	10	NR	1	p-ANCA 1:100	NR	NR	NR	NR	0.23	None	None	RRT	NA	NR	Concurrent	
Tse WY [33]	1997	UK	65	M	None	0	0	1.74	2.4	NR	1	p-ANCA 1:100	NR	NR	NR	NR	0.28	None	steroids, CPA	1.47	NA	NR	Concurrent	
Tse WY [33]	1997	UK	70	M	None	0	0	13.8	NA	NR	0	c-ANCA 1:25	NR	NR	NR	NR	0.73	ENT, Skin	steroids, AZA, CPA, PE	2.29	NA	NR	Concurrent	
Gao M [34]	2021	China	62	M	Pulmonary tuberculosis	0	0	0.86	7.09	0	1	p-ANCA; MPO 253.96 U/mL	-	NR	NR	NR	0	None	IV/PO steroids, MZR	0.69	0.13	NR	Concurrent	
Yu F [35]	2007	China	21	F	HTH	NR	NR	1.1	1.86	NR	1	MPO; human leukocyte elastase; cathepsin G	NR	NR	NR	NR	0.04	Skin	IV/PO steroids, CPA, PE, stop PTU	CR	CR	a	Concurrent	PTU-induced
Yu F [35]	2007	China	43	F	HTH	NR	NR	3.2	1.1	NR	1	MPO	NR	NR	NR	NR	0.04	E, L	IV/PO steroids, CPA, stop PTU	PR	PR	a	Concurrent	PTU-induced
Chen Y [36]	2014	China	43	F	HTH	0	0	0.71	1.25	NR	1	MPO 174 RU/ml; p-ANCA 1:160	NR	NR	NR	NR	0	None	PO steroids, MMF, stop PTU	0.6	0.18	b	Concurrent	PTU-induced
Chen Y [36]	2014	China	39	F	HTH	0	0	1.01	5.97	NR	1	PR3 264 RU/ml; p-ANCA 1:80	NR	NR	NR	NR	0	E, Skin	PO steroids, AZA, MMF, stop PTU	0.58	0.1	b	Concurrent	PTU-induced
Gaber L.W. [37]	1993	USA	64	M	None	0	0	1.6	1.6	1	1	c-ANCA >1:320	NR	NR	NR	IgG4 positive	0.11	E, ENT, L	PO steroids, PO CPA	1.6	Negative	NR	Concurrent	
Surindran S [38]	2012	USA	56	M	HT	0	0	13.4	14	1	1	p-ANCA 1:160; MPO 31.5 U/mL	+	NR	NR	NR	1	None	PO steroids, CPA, PE	2.3	4	NR	Concurrent	
Mathieson PW [39]	1996	UK	53	M	RA	0	1(RA)	4.05	2.7	1	1	p-ANCA	NR	NR	NR	NR	0.3	E, Skin	IV/PO steroids, AZA, IV/PO CPA	1.37	NR	NR	Concurrent	D-penicillamine-induced
Iwafuchi Y [40]	2013	Japan	71	M	HT	0	0	14.5	9	1	1	MPO 68.6 U/mL	NR	NR	NR	IgG4 positive	0.875	None	IV/PO steroids	RRT	RRT	NR	Concurrent	
Rajesh C [41]	2025	India	58	M	None	0	0	3.4	3.9	1	1	MPO >200 EU	NR	NR	NR	NR	0.73	None	IV/PO steroids, CPA, PE	2	NR	NR	Concurrent	
Rajesh C [41]	2025	India	51	F	None	0	0	1.4	7.8	1	1	MPO >200 EU	NR	-	NR	NR	0.8	None	IV/PO steroids, RTX	2.5	NR	NR	Concurrent	
Zhu Y [42]	2019	China	50	M	Eczema	0	0	0.87	6.4	0	1	PR3 1:10	+	NR	NR	IgG4 positive	0	ENT, L, Skin, BM	PO steroids, IV CPA	0.79	0.1	NR	Concurrent	
Balafa O [43]	2015	Greece	58	M	CHD	0	0	7.39	8	1	1	p-ANCA 1:160	NR	NR	NR	NR	0.42	None	IV/PO steroids, IV CPA, PE	1.51	1.73	NR	Concurrent	

Author	Year	Country	Age	Sex	Comorbidity	Malignancy	Autoimmune diseases other than ANCA(0/1)	Cre	PU	RPGN(0/1)	Hematuria	ANCA	PLA2R serum	PLA2R kidney	TSHD7A	IgG subclass	Crescent	Extrarenal lesions	Treatment	Outcome Cre	Outcome UP	Remission	Timing	Trigger/etiology
Ghandour M [44]	2022	USA	53	M	T2DM, HT	0	0	8.04	19.2	1	1	MPO	NR	-	kidney -	NR	0.5	ENT, L, N	IV/PO steroids, IV CPA	RRT	NA	NR	Concurrent	
Yoshida H [45]	2021	Japan	78	M	CVA, GU, HT, hyperuricemia, insomnia	0	0	1.41	1.66	1	1	MPO	NR	NR	NR	NR	0.62	L	IV/PO steroids, IV CPA	1.45	NA	NR	MN preceding	
Morimoto C [46]	2026	Japan	41	M	hyperuricemia	0	0	1.73	7.08	1	1	MPO 1340 U/ml	NR	+	NR	IgG4 dominant	0.55	None	IV/PO steroids, Avacopan, RTX	1.3	CR	NR	Concurrent	
De Souza LM [47]	2021	USA	62	M	None	0	0	NR	0.72	1	NR	c-ANCA 1:320; PR3 437.4	NR	NR	NR	NR	NR	None	NR	RRT	RRT	NR	MN preceding	
Angel Korman A [48]	2022	Israel	34	M	None	0	0	2.9	0.63	1	1	MPO 97 IU/ml	NR	-	NR	IgG4 not dominant	9%%	ENT	None	1.2	0.75	NR	Concurrent	G-CSF induced
Crowe AV [49]	1995	UK	68	M	None	0	0	13	2.2	1	NR	c-ANCA	NR	NR	NR	NR	NR	L	IV/PO steroids, AZA, IV CPA	RRT	RRT	NR	Concurrent	
Yedidag A [50]	1997	USA	61	M	Esophageal squamous cell carcinoma	1	0	normal range	NR	0	NR	p-ANCA	NR	NR	NR	NR	0	L	steroids	NR	NR	NR	Concurrent	
Rabie M [51]	2025	USA	68	F	HT, lupus nephritis features	0	1(lupus nephritis features)	NR	NR	1	NR	MPO 234; PR3 41.2	NR	NR	NR	NR	NR	None	Strroids, hydroxychloroquine, RTX	NR	NR	NR	Concurrent	hydralazine-induced
Chang A [52]	2007	USA	49	F	HT, SLE	0	1	2.8	4.47	1	1	p-ANCA; MPO 22 U/mL	NR	NR	NR	NR	0.63	None	IV steroids, IV CPA, MMF	1	1	NR	Concurrent	
Roca-Argente L [53]	2015	Spain	49	M	HCV	0	0	7.09	1.35	1	1	p-ANCA 1:1,280; MPO 56.4 U/mL; PR3 47.10 U/mL	NR	NR	NR	NR	+	Skin	IV/PO steroids	1.98	<1	NR	Concurrent	Cocaine use
Zhou GY [54]	2012	China	64	M	None	0	0	3.97	5.4	1	1	MPO 145 U/ml	NR	NR	NR	NR	0.47	None	IV/PO steroids, CPA	2.07	1.9	NR	Concurrent	
Nasr SH [55]	2009	USA	57	F	None	0	0	2.5	NA	1	NR	p-ANCA; MPO	NR	NR	NR	NR	0.29	L	PO steroids, IV CPA, MMF	RRT	NA	NR	Concurrent	
Nasr SH [55]	2009	USA	65	M	Polymyositis	0	1(Polymyositis)	2.2	5.8	1	NR	p-ANCA	NR	NR	NR	NR	0.05	None	PO steroids, PO CPA	1.8	Negative	NR	Concurrent	
Nasr SH [55]	2009	USA	37	F	None	0	0	3.5	5.5	1	NR	p-ANCA; MPO	NR	NR	NR	NR	0.16	None	IV/PO steroids, IV CPA	0.9	0.77	NR	Concurrent	
Nasr SH [55]	2009	USA	39	F	None	0	0	1.3	6.8	1	NR	c-ANCA; PR3	NR	NR	NR	NR	0.15	E	PO steroids, PO CPA	2.1	4.2	NR	Concurrent	
Nasr SH [55]	2009	USA	47	M	Gout	0	0	8.6	14	1	NR	p-ANCA; MPO	NR	NR	NR	NR	0.83	None	PO steroids, IV CPA	RRT	NA	NR	Concurrent	
Nasr SH [55]	2009	USA	71	M	None	0	0	3	6.6	1	NR	p-ANCA; MPO	NR	NR	NR	NR	0.59	None	PO steroids, PO CPA	2.8	0.817	NR	Concurrent	
Nasr SH [55]	2009	USA	50	M	None	0	0	0.9	0.8	0	NR	c-ANCA; PR3	NR	NR	NR	NR	0.15	L	PO steroids, AZA, PO CPA	0.8	Negative	NR	Concurrent	
Nasr SH [55]	2009	USA	62	F	Polymyositis, Mixed connective tissue disease	0	1(Polymyositis, MCTD)	4.1	16	1	NR	PR3; MPO	NR	NR	NR	NR	0.3	None	IV/PO steroids, IV CPA	RRT	NA	NR	Concurrent	
Nasr SH [55]	2009	USA	78	M	Prostate cancer	1	0	4	NA	1	NR	c-ANCA	NR	NR	NR	NR	0.33	None	PO steroids, IV CPA	2.1	0.701	NR	Concurrent	
Nasr SH [55]	2009	USA	79	F	None	0	0	8.9	NA	1	NR	ANCA	NR	NR	NR	NR	0.5	None	None	Died	NA	NR	Concurrent	
Nasr SH [55]	2009	USA	59	M	None	0	0	6	3.5	1	NR	c-ANCA; MPO	NR	NR	NR	NR	0.41	None	PO steroids, IV CPA	RRT	NA	NR	Concurrent	
Nasr SH [55]	2009	USA	69	M	Prostate cancer	1	0	5.4	1.9	1	NR	p-ANCA	NR	NR	NR	NR	0.19	None	PO steroids, IV CPA	3.5	1.1	NR	Concurrent	
Nasr SH [55]	2009	USA	51	M	None	0	0	8.7	5.8	1	NR	p-ANCA	NR	NR	NR	NR	0.24	None	IV/PO steroids, CPA	RRT	RRT	NR	Concurrent	
Nasr SH [55]	2009	USA	58	F	None	0	0	3.1	5	1	NR	c-ANCA; PR3	NR	NR	NR	NR	0.23	None	IV/PO steroids, CPA	0.8	1.2	NR	MN preceding	
Nikolopoulou A [56]	2019	UK	70	M	NA	0	0	10	3.35	1	1	MPO >134 u/ml	NR	NR	NR	NR	0.25	L	steroids, CPA	RRT	NA	NR	Concurrent	
Nikolopoulou A [56]	2019	UK	73	M	NA	0	0	3.95	37.8	1	1	MPO 86 u/ml	NR	NR	NR	NR	0.07	None	steroids, CPA, PE	RRT	NA	NR	Concurrent	
Nikolopoulou A [56]	2019	UK	66	M	NA	0	0	6.33	0.78	1	1	MPO >134 u/ml	NR	NR	NR	NR	0.28	None	steroids, CPA, RTX	2.53	0.08	NR	Concurrent	
Nikolopoulou A [56]	2019	UK	34	F	NA	0	0	0.97	18.7	1	1	MPO 19 u/ml	NR	NR	NR	NR	0.08	None	steroids, MMF	0.9	0.87	NR	Concurrent	
Nikolopoulou A [56]	2019	UK	74	F	NA	0	0	2.45	9.3	1	1	MPO 78 u/ml	NR	NR	NR	NR	0.29	L	steroids, CPA, RTX	1.06	0.89	NR	Concurrent	
Nikolopoulou A [56]	2019	UK	73	F	NA	0	0	2.01	4.94	1	1	MPO 74 u/ml	NR	NR	NR	NR	0.06	None	steroids, MMF	1.87	0.44	NR	Concurrent	
Nikolopoulou A [56]	2019	UK	64	M	NA	0	0	2.98	0.99	1	1	PR3 544 u/ml	NR	NR	NR	NR	0.33	L	steroids, CPA, RTX	0.94	0.55	NR	Concurrent	
Granata A [57]	2011	Italy	67	M	HT	0	0	NR	4.8	1	1	p-ANCA 1:40	NR	NR	NR	NR	0.27	None	IV/PO steroids, CPA	1.6	1.2	NR	Concurrent	
Suwabe T [58]	2005	Japan	68	F	None	0	0	3	6.97	1	1	MPO 204IU/ml	NR	NR	NR	IgG4 dominant	0.69	None	PO steroids, IV CPA	1.8	0.5	NR	Concurrent	
Erez D [59]	2022	Israel	67	F	HT	0	1(SLE)	1.15	1.3	1	0	p-ANCA 1:40	NR	NR	NR	NR	0.5	None	IV/PO steroids, CPA, AZA	0.9	0.15	NR	Concurrent	
Dwyer K.M [60]	2001	Australia	67	M	HT	0	0	0.69	7.16	1	1	p-ANCA 1:160; MPO >100	NR	NR	NR	NR	+	None	PO steroids, AZA	RRT	NR	NR	Concurrent	
Dwyer K.M [60]	2001	Australia	69	M	None	0	0	0.13	0.56	1	1	p-ANCA; MPO 78 U/ml	NR	NR	NR	NR	0.23	None	PO steroids, PO CPA	0.11	NR	NR	Concurrent	
Ridder RM [61]	2005	Germany	12	F	ulcerative colitis	0	1(ulcerative colitis)	0.41	1	0	1	PR3 8 U/l; p-ANCA 1:1,280	NR	NR	NR	NR	0	None	PO steroids, AZA	NR	NR	NR	Concurrent	
Ozdemir N [62]	2006	Turkey	10	M	atopic dermatitis, short stature, spondylo-epiphyseal dysplasia,HT	0	0	NR	3.41	0	NR	p-ANCA	NR	NR	NR	NR	0	None	PO steroids	NR	3.41	NR	Concurrent	
Ram R [63]	2014	India	37	M	None	0	0	1.9	8.7	1	1	p-ANCA; MPO	NR	NR	NR	NR	0.33	ENT, L, N, Skin	IV/PO steroids, IV CPA	1.2	0.2	NR	Concurrent	
Moinuddin I [64]	2016	USA	50	F	Asthma, HT, RA	0	1(RA)	2.2	1.6	1	1	p-ANCA; MPO 134 U/ml	-	-	NR	IgG4 negative	+	Skin	IV/PO steroids, AZA, PO CPA	0.8	0.11	NR	Concurrent	Cocaine use
Kanodia K [65]	2014	India	48	M	HT	0	0	9.13	NA	1	1	MPO 220 U/ml	NR	NR	NR	NR	0.36	None	IV/PO steroids, IV CPA	RRT	NA	NR	Concurrent	
Yoshida S [66]	2021	Japan	73	F	HT, DLP	0	0	0.91	2.6	1	1	PR3 725 U/mL	-	NR	NR	IgG4 positive	0.04	E, L	PO steroids, RTX	0.5-1.0	0.15	NR	Concurrent	
Matsumoto K [8]	2009	Japan	NA	F	HT, DLP	0	0	0.6	1.6	0	1	MPO >640 U/ml	NR	NR	NR	IgG4 dominant	0.3	None	IV/PO steroids, PO CPA	around 1	Negative	NR	Concurrent	
Tsuruoka K [67]	2008	Japan	72	F	HTH, T2DM, HT	0	0	3.62	2.2	1	1	MPO 68 EU	NR	NR	NR	NR	0.12	None	IV/PO steroids, stop MMI	RRT	NA	NR	Concurrent	MMI-induced
Quarenghi MI [68]	1998	Italy	52	M	Psoriatic arthritis, psoriasis	0	1(psoriasis)	1.03	5.2	1	1	p-ANCA; MPO 570 UI/ml	NR	NR	NR	NR	+	None	IV/PO steroids, CPA	1.36	NR	NR	Concurrent	Gold-salt–induced
Kanahara K [69]	1997	Japan	47	F	None	0	0	1.1	4.9	1	1	MPO 297 EU	NR	NR	NR	NR	+	L	steroids, AZA	NR	NR	NR	MN preceding	
Manabe S [70]	2017	Japan	19	M	None	0	0	1.2	3	1	1	MPO 230 IU/l	NR	-	NR	IgG4 not dominant	0.14	ENT, L, N, RetP	IV/PO steroids, IV CPA	RRT	NR	NR	Concurrent	
Tominaga K [9]	2024	Japan	63	F	NR	0	0	NA	2.65	NR	NR	MPO 25.6 U/ml	NR	NR	NR	NR	0.11	NR	PO steroids	NR	0.13	NR	ANCA preceding	

Author	Year	Country	Age	Sex	Comorbidity	Malignancy	Autoimmune diseases other than ANCA(0/1)	Cre	PU	RPGN(0/1)	Hematuria	ANCA	PLA2R serum	PLA2R kidney	TSHD7A	IgG subclass	Crescent	Extrarenal lesions	Treatment	Outcome Cre	Outcome UP	Remission	Timing	Trigger/etiology
Tominaga K [9]	2024	Japan	77	F	NR	0	0	NA	3.91	NR	NR	MPO 21 U/ml	NR	NR	NR	NR	0.54	NR	PO steroids	NR	1.81	NR	Concurrent	
Tominaga K [9]	2024	Japan	64	F	NR	0	0	NA	2.35	NR	NR	MPO 64.3 U/ml	NR	NR	NR	NR	0.5	NR	IV/PO steroids	NR	1.29	NR	Concurrent	
Tominaga K [9]	2024	Japan	73	M	NR	0	0	NA	3.04	NR	NR	MPO 20.1 U/ml	NR	NR	NR	NR	0.37	NR	IV/PO steroids	NR	0.28	NR	Concurrent	
Tominaga K [9]	2024	Japan	60	F	NR	0	0	NA	1.58	NR	NR	MPO 80.5 U/ml	NR	NR	NR	NR	0.58	NR	IV/PO steroids, AZA, CPA	NR	0.68	NR	Concurrent	
Tominaga K [9]	2024	Japan	34	F	NR	0	0	NA	6.8	NR	NR	MPO 245 U/ml	NR	NR	NR	NR	0.63	NR	IV/PO steroids, MMF	NR	1.26	NR	Concurrent	
Tominaga K [9]	2024	Japan	52	M	NR	0	0	NA	9.42	NR	NR	MPO 19.4 U/ml	NR	NR	NR	NR	0.61	NR	PO steroids, AZA, MZR	NR	1.74	NR	ANCA preceding	
Carrara C [71]	2016	Italy	34	F	None	0	0	4.2	2.4	1	1	p-ANCA; MPO > 311.4 U	NR	-	NR	NR	0.33	Skin	IV/PO steroids, PE, RTX	1.6	NR	NR	Concurrent	Cocaine use
Bhatnagar M [72]	2023	USA	52	F	HT	0	1(SLE)	1.66	NR	1	1	PR3 24.1 units/mL; c-ANCA 1:640; MPO 38.9 units/mL	NR	NR	NR	NR	0.52	L	steroids, CPA, PE	RRT	NR	NR	Concurrent	hydralazine-induced
Burkhart R [73]	2015	USA	79	M	HT, DLP, hypothyroidism, gout, fatty liver, ILD, mitral stenosis, pulmonary HT	0	1(SLE)	9.2	3	1	1	p-ANCA 1:640; MPO 657 AU/ml	NR	-	NR	IgG4 negative	0.38	L	IV/PO steroids, PE, RTX	RRT	NR	NR	MN preceding	
Chen YX [74]	2007	China	43	F	HTH	0	NR	0.59	10.6	NR	1	MPO 1:320, 68.35 RU/ml	NR	NR	NR	NR	0	None	PO steroids, stop PTU	Remission	Remission	c	Concurrent	PTU-induced
Nishio S [75]	2001	Japan	40	F	HTH	0	0	1.8	NR	1	1	MPO 563 EU	NR	NR	NR	NR	0	None	PO steroids, CPA, stop PTU	NR	NR	NR	MN preceding	PTU-induced
Yamazaki T [76]	2023	Japan	78	M	SLE	0	1(SLE)	4.25	2	1	1	MPO 9.7 U/ml	-	-	NR	IgG4 positive	0.57	None	IV/PO steroids, PE	RRT	RRT	NR	Concurrent	
Bao D [77]	2025	China	74	M	RA, COPD	0	1(RA)	11.87	1.51	1	1	MPO 169.8 RU/mL	+	NR	NR	NR	NR	None	IV/PO steroids, CPA	RRT	RRT	NR	MN preceding	
Gupta A [78]	2012	Canada	67	F	T2DM, HT	0	1(SLE)	2.32	1.18	1	1	p-ANCA; MPO 100 IU/ml	-	NR	NR	NR	0.5	None	IV/PO steroids, CPA	1.18	0.69	NR	Concurrent	
Golas VL [79]	2020	USA	58	M	NR	NR	NR	3.12	NR	1	NR	p-ANCA	NR	NR	NR	NR	0.10	NR	NR	4.4	NR	NR	Concurrent	
Golas VL [79]	2020	USA	79	M	NR	NR	NR	7.55	NR	1	NR	ANCA; MPO	NR	NR	NR	NR	0.40	NR	NR	RRT	NR	NR	Concurrent	

- Remission codes refer to author-reported remission definitions at the study level, not to whether an individual case achieved remission.
- a: Yu F 2007. Complete remission: (1) normalization of renal function if renal function abnormality existed before and (2) disappearance of hematuria, proteinuria, and extrarenal manifestations of systemic vasculitis. Partial remission: (1) stabilization or improvement of renal function if renal function abnormality existed before or dialysis independent if dialysis dependent before, and/or (2) resolution of hematuria and proteinuria and/or resolution of extrarenal manifestations of systemic vasculitis.
- b: Chen Y 2014. Complete remission: normal renal function and normal urine analysis, with no extrarenal manifestations of systemic vasculitis. Partial remission: stabilization or improvement in renal function if renal dysfunction existed, or independence from dialysis if the patient had been dialysis dependent at the beginning of the treatment, with or without resolution of hematuria and proteinuria and extrarenal manifestations of systemic vasculitis.
- c: Chen YX 2007. Remission was defined as stabilization or improvement of renal function, resolution of hematuria, and resolution of extrarenal manifestations of systemic vasculitis. Persistence of proteinuria was not considered indicative of persistence of disease activity.
- NR: remission definition not reported.

Values at diagnosis are presented for Cre (in mg/dL) and PU (in g/gCr or g/day). Malignancy was coded as present (1) or absent (0). Autoimmune diseases other than ANCA were coded as present (1) or absent (0); when present, the specific disease is shown in parentheses when available. RPGN was coded as present (1) when the clinical course described in the original report was judged to meet a rapidly progressive decline in kidney function, and absent (0) otherwise. Hematuria is indicated as present (1) or absent (0). ANCA results are shown as reported in the original articles; assay type, units, and positivity thresholds were not standardized across reports. For PLA2R serum, PLA2R kidney, and THSD7A, + and - indicate positive and negative results, respectively, and NR indicates not reported. PLA2R kidney refers to tissue staining. Crescent formation is indicated as present (1), absent (0), or by the percentage of glomeruli with crescents if available. Outcome Cre and Outcome UP represent the latest reported post-treatment values. When numeric values were unavailable, author-reported outcomes such as RRT, remission, or death were retained. Timing was categorized as Concurrent, MN preceding, or ANCA preceding; Concurrent indicates diagnoses made within 6 months of each other, whereas MN preceding and ANCA preceding indicate intervals of >6 months. Data on non-specific systemic symptoms, such as general malaise, are not included in this table.

Abbreviations: AZA; Azathioprine, BM; Bone marrow, BPH; benign prostatic hypertrophy, BPI; bactericidal permeability increasing, CHD; Coronary heart disease, COPD; Chronic obstructive pulmonary disease, CPA; Cyclophosphamide, CR; complete remission, Cre; Creatinine, CVA; Cerebrovascular accident, CyA; Ciclosporin, DLP; Dyslipidemia, E; Eyes, ENT; Ear and/or nose and/or throat, G-CSF; Granulocyte colony-stimulating factor, GI; Gastrointestinal, GU; Gastric ulcer, HCV; Hepatitis C Virus, HT; Hypertension, HTH; Hyperthyroidism, ID; Intellectual disability, ILD; Interstitial lung disease, IV; Intravenous, L; Lungs, MGUS; Monoclonal gammopathy of undetermined significance, MMF; Mycophenolate mofetil, MMI; Methimazole, MZR; Mizoribine, NA; Not available, N; Nerves, PE; Plasma exchange, PLA2R; phospholipase A2 receptor, PO; By mouth, PR; partial remission, PTU; Propylthiouracil, PU; Proteinuria, RA; Rheumatoid arthritis, RetP; Retroperitoneum, RRT; Renal replacement therapy, RTX; Rituximab, SSS; Sick sinus syndrome, T2DM; Type 2 diabetes, TAC; Tacrolimus, UK; United Kingdom, USA; United States of America.

Supplementary References

13. Nakada Y, Tsuboi N, Takahashi Y, Yoshida H, Hara Y, Okonogi H, et al. A case of concurrent MPO-/PR3-negative ANCA-associated glomerulonephritis and membranous glomerulopathy. Case Rep Nephrol. 2015;2015:316863.
14. Kim YH, Kim HR, Ham YR, Jeon JW, Chung S, Choi DE, et al. A case of membranous glomerulonephritis with superimposed anti-neutrophil cytoplasmic antibody-associated rapidly progressive crescentic glomerulonephritis. Chonnam Med J. 2015;51:102-5.
15. Matsuda J, Nagayama I, Yamaguchi Y, Itano S, Mori D, Imakita N, et al. A case of crescentic poststreptococcal acute glomerulonephritis (PSAGN) accompanied by membranous nephropathy. Nihon Jinzo Gakkai Shi. 2013;55:567-73.
16. Hu ZJ, Niu K, Liu B, Shi YN. A case of membranous nephropathy and myeloperoxidase anti-neutrophil cytoplasmic antibody-associated glomerulonephritis. Exp Ther Med. 2014;8:1170-2.
17. Mahmood SB, Ahmad H, Wu J, Haselby D, LeClaire MM, Nasr R. Anti-neutrophil cytoplasmic antibody-positive eosinophilic granulomatosis with polyangiitis: can it cause membranous nephropathy? Scand J Rheumatol. 2019;48:256-7.
18. Fujii T, Kawamata T, Ueda S, Akikusa B, Hasegawa S, Tsukahara T, et al. A case of membranous nephropathy with ANCA-associated necrotizing glomerulonephritis during oral administration of PTU for Graves' disease. Nihon Jinzo Gakkai Shi. 2003;45:771-7.
19. Shimada M, Fujita T, Nakamura N, Narita I, Shimaya Y, Murakami R, et al. A case of myeloperoxidase anti-neutrophil cytoplasmic antibody (MPO-ANCA)-associated glomerulonephritis and concurrent membranous nephropathy. BMC Nephrol. 2013;14:73.
20. Uyama S, Ohashi N, Iwakura T, Ono M, Fujikura T, Sakao Y, et al. A case presenting with the possible relationship between myeloperoxidase-antineutrophil cytoplasmic antibody-associated glomerulonephritis and membranous changes of the glomerular basement membrane. CEN Case Rep. 2013;2:53-8.
21. Takahashi N, Kimura H, Kitai R, Sato M, Yoneda M, Yamamoto C, et al. Acute on chronic subdural hematoma as a rare complication in a microscopic polyangiitis patient receiving antithrombotic treatment. Clin Nephrol. 2009;72:211-5.
22. Collister D, Sathianathan C, Ryz K, Karpinski M, Bernstein K, Gibson IW. ANCA associated vasculitis secondary to levamisole-adultered cocaine with associated membranous nephropathy: a case series. Am J Nephrol. 2017;45:209-16.
23. Sekine S, Yumura W, Tanaka Y, Suganuma S, Onuki T, Uchida K, et al. An elderly case of ANCA-positive membranous nephropathy. Nihon Jinzo Gakkai Shi. 2002;44:44-9.
24. Meisels IS, Stillman IE, Kuhlik AB. Anti-glomerular basement membrane disease and dual positivity for antineutrophil cytoplasmic antibody in a patient with membranous nephropathy. Am J Kidney Dis. 1998;32:646-8.
25. Etter C, Gaspert A, Regenass S, Wuthrich RP, Kistler T, Kain R, et al. Anti-hLAMP2-antibodies and dual positivity for anti-GBM and MPO-ANCA in a patient with relapsing pulmonary-renal syndrome. BMC Nephrol. 2011;12:26.
26. Kakita T, Nagatoya K, Takimoto H, Matsuda H, Mori T, Kawaguchi A, et al. Antineutrophil cytoplasmic antibody-associated crescentic glomerulonephritis with membranous nephropathy treated using thiamazole. CEN Case Rep. 2013;2:165-9.
27. Tominaga K, Uchida T, Imakiire T, Itoh K, Shimazaki H, Nakanishi K, et al. Anti-neutrophil cytoplasmic antibody-associated glomerulonephritis with detection of myeloperoxidase and phospholipase A2 receptor in membranous nephropathy-lesions: report of two patients with microscopic polyangiitis. BMC Nephrol. 2018;19:120.
28. Jiang S, Zhuo L, Zou G, Gao H, Li W. Anti-neutrophil cytoplasmic antibody seropositivity in young adults aged up to 35 years: kidney histopathological findings and patient outcomes. J Int Med Res. 2022;50:3000605221078097.
29. Zuo Y, Cavalcante LB, Smelser JM, Sanghani N, Dwyer JP, Lewis JB, et al. Anti-phospholipase A2 receptor in nonlupus patients with membranous nephropathy and crescents. Glomerular Dis. 2021;2:75-82.
30. Watanabe S, Arimura Y, Nomura K, Kawashima S, Yoshihara K, Kaname S, et al. A case of MPO-ANCA-associated vasculitis with membranous nephropathy. Nihon Jinzo Gakkai Shi. 2011;53:46-52.
31. P'ng CH, Dear L, Phoon R, Brown D. A rare case of concurrent myeloperoxidase anti-neutrophil cytoplasmic antibody (MPO-ANCA)-associated necrotising and crescentic glomerulonephritis and membranous glomerulonephritis. Pathology. 2017;49:100-3.
32. Fang W, Li K. A rare case of overlap syndrome: ANCA-associated glomerulonephritis with membranous nephropathy and IgA deposition. Clin Nephrol. 2025;104:156-8.
33. Tse WY, Howie AJ, Adu D, Savage CO, Richards NT, Wheeler DC, et al. Association of vasculitic glomerulonephritis with membranous nephropathy: a report of 10 cases. Nephrol Dial Transplant. 1997;12:1017-27.
34. Gao M, Wang J, Dong D, Liu Y, Sun W, Xu Z, et al. Coexistence of antineutrophil cytoplasmic antibody-associated vasculitis and membranous nephropathy: a case report. Ann Palliat Med. 2021;10:5875-80.
35. Yu F, Chen M, Gao Y, Wang SX, Zou WZ, Zhao MH, et al. Clinical and pathological features of renal involvement in propylthiouracil-associated ANCA-positive vasculitis. Am J Kidney Dis. 2007;49:607-14.
36. Chen Y, Bao H, Liu Z, Zhang H, Zeng C, Liu Z, et al. Clinico-pathological features and outcomes of patients with propylthiouracil-associated ANCA vasculitis with renal involvement. J Nephrol. 2014;27:159-64.
37. Gaber LW, Wall BM, Cooke CR. Coexistence of anti-neutrophil cytoplasmic antibody-associated glomerulonephritis and membranous glomerulopathy. Am J Clin Pathol. 1993;99:211-5.
38. Surindran S, Ayalon R, Hasan N, Beck Jr LH, Salant DJ, Barisoni L, et al. Coexistence of ANCA-associated glomerulonephritis and anti-phospholipase A(2) receptor antibody-positive membranous nephropathy. Clin Kidney J. 2012;5:162-5.
39. Mathieson PW, Peat DS, Short A, Watts RA. Coexistent membranous nephropathy and ANCA-positive crescentic glomerulonephritis in association with penicillamine. Nephrol Dial Transplant. 1996;11:863-6.
40. Iwafuchi Y, Morita T, Oyama Y, Kamimura A, Miyazaki S, Narita I. Combined membranous nephropathy and crescentic glomerulonephritis with concurrent anti-glomerular basement membrane antibody and myeloperoxidase-specific anti-neutrophil cytoplasmic antibody. CEN Case Rep. 2013;2:107-11.
41. Rajesh C, Mishra U, Roy S, Alam R, Raj Mani SS, Eapen JJ, et al. Concomitant histological features of membranous nephropathy and anti-neutrophil cytoplasmic antibody associated vasculitis. Indian J Nephrol. 2025;35:105-8.
42. Zhu Y, Chang Q, Cao X, Zheng S, Li P, Luan J, et al. Co-occurrence of PLA2R-positive membranous nephropathy without crescents, and PR3-positive eosinophilic granulomatosis with polyangiitis. Clin Nephrol. 2019;92:208-15.
43. Balafa O, Kalaitzidis R, Liapis G, Xiromeriti S, Zarzoulas F, Baltatzis G, et al. Crescentic glomerulonephritis and membranous nephropathy: a rare coexistence. Int Urol Nephrol. 2015;47:1373-7.
44. Ghandour M, Osman H, Alkassis S, Charles A, Zalewski K, Weinberger J, et al. Crescentic glomerulonephritis and membranous nephropathy: a rare overlap. Case Rep Nephrol. 2022;2022:8292458.
45. Yoshida H, Takahashi N, Horiguchi T, Yasuhara H, Tanaka T, Chen Y, et al. Development of myeloperoxidase anti-neutrophil cytoplasmic antibody-positive necrotizing crescentic glomerulonephritis in an elderly patient with immunological kidney disease. Intern Med. 2021;60:3927-35.
46. Morimoto C, Fujigaki Y, Anzai H, Terakawa R, Hiraike K, Asakawa S, et al. Dual MPO-ANCA and PLA2R positivity in crescentic glomerulonephritis with MPO-associated membranous nephropathy: a case report with pathogenic insights. CEN Case Rep. 2026;15:21.
47. De Souza LM, Ghahramani N, Abendroth C, Kaur G. Evaluating a living donor with rheumatoid arthritis for a recipient with end-stage renal disease from antineutrophil cytoplasmic antibodies associated vasculitis. Cureus. 2021;13:e18117.
48. Angel Korman A, Leiba A, Edel Y, Rapoport V, Tobar A, Leiba M. G-CSF-induced ANCA associated glomerulonephritis in the presence of silent membranous "full house nephropathy" in an altruistic bone marrow donor. Ren Fail. 2022;44:1498-1500.
49. Crowe AV, Woolfson RG, Griffiths MH. Glomerulocystic kidney disease associated with Wegener's granulomatosis and membranous glomerulonephritis: a case report. Nephrol Dial Transplant. 1995;10:888-90.
50. Yedidag A, Zikos D, Spargo B, MacEntee P, Berkelhammer C. Esophageal carcinoma presenting with nephrotic syndrome: association with anti-neutrophil cytoplasmic antibody. Am J Gastroenterol. 1997;92:326-8.
51. Rabie M, Scalise M. Hydralazine-induced ANCA-associated vasculitis with lupus nephritis features and a unique antibody profile. J Community Hosp Intern Med Perspect. 2025;15:58-60.
52. Chang A, Aneziokoro O, Meehan SM, Quigg RJ. Membranous and crescentic glomerulonephritis in a patient with anti-nuclear and anti-neutrophil cytoplasmic antibodies. Kidney Int. 2007;71:360-5.
53. Roca-Argente L, Moll-Guillen JL, Espi-Reig J, Blanes-Julia J, Garcia-Martinez AM, Pujol-Marco C, et al. Membranous glomerulonephritis and cellular crescents induced by levamisole-adulterated cocaine abuse: a case report. Ann Transl Med. 2015;3:271.
54. Zhou GY. Membranous glomerulonephritis associated with myeloperoxidase anti-neutrophil cytoplasmic antibody-associated glomerulonephritis. Nefrologia. 2012;32:4.
55. Nasr SH, Said SM, Valeri AM, Stokes MB, Masani NN, D'Agati VD, et al. Membranous glomerulonephritis with ANCA-associated necrotizing and crescentic glomerulonephritis. Clin J Am Soc Nephrol. 2009;4:299-308.
56. Nikolopoulou A, Huang-Doran I, McAdoo SP, Griffith ME, Cook HT, Pusey CD. Membranous glomerulonephritis with crescents. Kidney Int Rep. 2019;4:1577-84.
57. Granata A, Floccari F. Membranous glomerulonephritis with superimposed ANCA-associated vasculitis: another case report. NDT Plus. 2011;4:80-1.
58. Suwabe T, Ubara Y, Tagami T, Sawa N, Hoshino J, Katori H, et al. Membranous glomerulopathy induced by myeloperoxidase-anti-neutrophil cytoplasmic antibody-related crescentic glomerulonephritis. Intern Med. 2005;44:853-8.
59. Erez D, Dovrish Z, Zahavi T, Cohen-Hagai K, Korzets Z. Membranous lupus nephritis and antineutrophil cytoplasmic antibody-associated vasculitis overlap syndrome: a case report. Isr Med Assoc J. 2022;24:268-70.
60. Dwyer KM, Agar JW, Hill PA, Murphy BF. Membranous nephropathy and anti-neutrophil cytoplasmic antibody-associated glomerulonephritis: a report of 2 cases. Clin Nephrol. 2001;56:394-7.
61. Ridder RM, Kreth HW, Kiss E, Grone HJ, Gordjani N. Membranous nephropathy associated with familial chronic ulcerative colitis in a 12-year-old girl. Pediatr Nephrol. 2005;20:1349-51.
62. Ozdemir N, Alpay H, Bereket A, Bereket G, Biyikli N, Aydogan M, et al. Membranous nephropathy in Schimke immuno-osseous dysplasia. Pediatr Nephrol. 2006;21:870-2.
63. Ram R, Rao CS, Swarnalatha G, Sireesha K, Madhuri HR, Rajasekhar L, et al. Membranous nephropathy superimposed on Churg-Strauss syndrome. Indian J Nephrol. 2014;24:57-9.
64. Moinuddin I, Madhrira M, Bracamonte E, Thajudeen B, Sussman A. Membranous nephropathy with crescents associated with levamisole-induced MPO-ANCA vasculitis. Pathol Res Pract. 2016;212:650-3.
65. Kanodia K, Vanikar A, Patel R, Suthar K, Nigam L, Kute V, et al. Membranous nephropathy with MPO-ANCA-associated crescentic GN. Nephrourol Mon. 2014;6:e20701.
66. Yoshida S, Hanai S, Nakagomi D, Kobayashi K, Takahashi K, Furuya F. Membranous nephropathy with proteinase 3-ANCA-associated vasculitis successfully treated with rituximab. Intern Med. 2021;60:145-50.
67. Tsuruoka K, Sekiya S, Yokoyama T, Koitabashi K, Shimazaki M, Sakurada T, et al. MPO-ANCA related vasculitis complicating mucinous cystadenoma of the pancreas and severe acute pancreatitis after steroid pulse therapy: a case report. Nihon Jinzo Gakkai Shi. 2008;50:948-53.
68. Quarenghi MI. MPO antibody-positive vasculitis in a patient with psoriatic arthritis and gold-induced membranous glomerulonephritis. Nephrol Dial Transplant. 1998;13:2104-6.
69. Kanahara K, Yorioka N, Nakamura C, Kyuden Y, Ogata S, Taguchi T, et al. Myeloperoxidase-antineutrophil cytoplasmic antibody-associated glomerulonephritis with membranous nephropathy in remission. Intern Med. 1997;36:841-6.
70. Manabe S, Hatano M, Nakano M, Fujii T, Nitta K, Nagata M. Myeloperoxidase-antineutrophil cytoplasmic antibody causes different renal diseases by immune-complex formation and pauci-immune mechanism: a case report. Pathol Int. 2017;67:419-24.
71. Carrara C, Emili S, Lin M, Alpers CE. Necrotizing and crescentic glomerulonephritis with membranous nephropathy in a patient exposed to levamisole-adulterated cocaine. Clin Kidney J. 2016;9:234-8.
72. Bhatnagar M, Agaronov A, Sarkisyan E, Sotoudeh Deilamy I, Pepito D, Akhondi H. Overlapping drug-induced vasculitis, ANCA-associated vasculitis, and lupus nephritis caused by low-dose hydralazine. Int J Rheum Dis. 2023;26:2272-7.
73. Burkhart R, Shah N, Abel M, Oliver JD 3rd, Lewin M. Pauci-immune necrotizing and crescentic glomerulonephritis with membranous lupus nephritis, fifteen years after initial diagnosis of secondary membranous nephropathy. Case Rep Nephrol. 2015;2015:120762.

74. Chen YX, Yu HJ, Ni LY, Zhang W, Xu YW, Ren H, et al. Propylthiouracil-associated antineutrophil cytoplasmic autoantibody-positive vasculitis: retrospective study of 19 cases. J Rheumatol. 2007;34:2451-6.
75. Nishio S, Shibazaki S, Yamamura T, Sasaki N, Yamada K, Kawata T, et al. Propylthiouracil induced ANCA-associated nephritis occurred in the course of membranous nephropathy. Nihon Naika Gakkai Zasshi. 2001;90:332-4.
76. Yamazaki T, Takahashi H, Takeuchi K, Sakamoto E, Tominaga K, Sakurabayashi S, et al. Rare case of exostosin 1/exostosin 2-related membranous lupus nephritis concomitant with dual ANCA- and anti-GBM antibody-associated crescentic glomerulonephritis effectively diagnosed by mass spectrometry: a case report. BMC Nephrol. 2023;24:218.
77. Bao D, Cui Z, Yao Y, Lu M, Zheng D, Wang Y. Rare case report: a man with rheumatoid arthritis developed membranous nephropathy and ANCA-associated vasculitis successively. BMC Nephrol. 2025;27:175.
78. Gupta A, Biyani M, Robertson SJ. Renal cocktail: too hard for a diabetic. Int Urol Nephrol. 2012;44:1289-92.
79. Golas VL, Lao KM, Misuraca MS, Li W, Marrone MG, Kanaan HD, Zhang PL. The clinical features of overlap syndrome (ANCA-associated crescentic glomerulonephritis [AACGN] and immune complex-mediated glomerulonephritis) are similar to those of AACGN alone. Int Urol Nephrol. 2021;53:515-21.

Title: Coexistence of ANCA-Associated Glomerulonephritis and Membranous Nephropathy: A Scoping Review Journal name: Clinical and Experimental Nephrology Author names: Ryosuke Saiki, Kan Katayama, Mutsuki Mori, Kaoru Dohi Affiliation: Department of Cardiology and Nephrology, Mie University Graduate School of Medicine E-mail: ryosuke-s@med.mie-u.ac.jp
